# Multiple Dimensions of Sweet Taste Perception Altered after Sleep Curtailment

**DOI:** 10.3390/nu11092015

**Published:** 2019-08-27

**Authors:** Edward J. Szczygiel, Sungeun Cho, Robin M. Tucker

**Affiliations:** Department of Food Science and Human Nutrition, Michigan State University, East Lansing, MI 48824, USA

**Keywords:** sleep curtailment, sweetness, hedonics, sweet liking phenotype, hierarchical cluster analysis

## Abstract

Short sleep duration increases preferences for high-carbohydrate and high-fat foods. It is unclear if insufficient sleep-induced changes in food preference are mediated by changes in taste perception and if these changes are related to sweetener type (sucrose or sucralose) or sweet liking phenotype. The primary objective of this study was to determine if sleep curtailment results in changes in sweet taste perception after sleep curtailment. Forty participants used a single-channel electroencephalograph to record both a habitual and curtailed night (33% reduction) of sleep at home. The following morning, multiple dimensions of sweet taste perception were measured, including preferred sweetener concentrations, patterns of sweet liking, and intensity perception over a range of concentrations. After curtailment, a significant increase in preferred concentration for both sucrose and sucralose (*p* < 0.001 for both) was observed. The slope of sucrose sweet liking increased after curtailment (*p* = 0.001). The slope of sucralose liking also increased, but this was not significant (*p* = 0.129). Intensity perception of the sweeteners was not altered by curtailment. Hierarchical cluster analysis was used to classify participants by sweet liking phenotype. Phenotypes were found to predict preferred sweetener concentration. These findings illustrate a possible need to control for sleep in food sensory studies and suggest a potential mechanism by which insufficient sleep can lead to excess energy intake.

## 1. Introduction

Nearly 40% of US adults report habitually sleeping less than the recommended 7 h per night [1], a proportion that has been steadily rising across all age groups since the 1980s [2]. Short sleep duration has routinely been associated with excess energy intake, weight gain, and obesity [3]. The relationship between insufficient sleep and excess energy intake is hypothesized to be motivated by both homeostatic [4,5,6,7,8] and hedonic [9,10,11,12] drives to eat. However, several recent studies suggest that hedonic drivers of food intake may predominate when sleep is insufficient [13,14,15,16,17]. For example, experiments using an ad libitum feeding paradigm have demonstrated that sleep curtailment increases energy intake, even when appetite-stimulating hormones are not elevated [18,19], suggesting that the relationship between insufficient sleep and excess energy intake is driven more by hedonic rather than homeostatic factors [15]. Further, when a meal is provided to minimize caloric deficit after sleep curtailment, individuals maintain an increased desire for excess intake from snacks [20], suggesting that changes in food reward processing after curtailment are not driven exclusively by hunger. Because hedonic evaluation of foods and beverages is based on sensory input from gustatory, olfactory, and somatosensory systems [21], altered sensory perception after short sleep may contribute to changes in food choice. Based on previous observational work reporting correlations between sleep duration and sweetness perception [22,23], the primary objective of the current study was to determine if sleep curtailment resulted in changes in preferred sweetener concentration and sweet taste intensity perception after sleep curtailment.

Sweetness is an ideal taste to begin to study the relationship between sleep and taste function for several reasons. First, nutritive sweeteners, such as sucrose, can contribute to excess energy intake and the development of obesity [24]. Second, sweet taste represents a palatable taste that will interact with brain reward systems; reward systems that are altered by insufficient sleep [25]. Brain imaging studies have demonstrated that insufficient sleep results in amplified reward from positive experiences [26] and increased positive hedonic perception of food cues [10,11,27,28,29]. These studies suggest that insufficient sleep results in increased reward sensitivity, which could lead to increased consumption of palatable food for pleasure (hedonic eating). Highly palatable food tends to be energy-dense, and therefore, increased hedonic eating can lead to excess energy intake [30]. While it is unclear if sleep-related changes in the hedonic perception of food are mediated by changes in taste perception, the preponderance of available evidence [22,23,31,32,33,34] suggests that insufficient sleep influences both taste function [31,33] and taste preference [22,23,32]. Altered sweet taste perception after a night of insufficient sleep may contribute to the link between insufficient sleep and excess energy intake, but more research is needed to confirm this. 

While sweetness is palatable, individuals differ in their hedonic responses to sweetness across a range of sweetener concentrations [35]. Three fundamental patterns of sweet liking have been repeatedly identified across studies [35,36,37]: sweet likers, who show an increase in liking as sweetener concentration increases; sweet dislikers, who show a decrease in liking as sweetener concentration increases; and “inverted U-shape” responders, who like sweetness up to a certain concentration and then begin to dislike subsequently higher concentrations, such that the pattern appears as an inverted U-shape. Additionally, a fourth phenotype has been reported, where the pattern of liking is stable over a range of concentrations [37]; however, many studies do not report observing this phenotype (for example: [38,39,40]). These fundamental patterns are referred to as sweet liking phenotypes, due to the fact that they are determined by both genetic and environmental factors [41,42,43]. The sweet liker phenotype has been found to be a meaningful predictor of several behaviors and traits, such as predicting the extent to which sweet taste from saccharin would condition hedonic response to a novel odorant when tasted together in solution [44], predicting the strength of positive emotional response to highly sweet samples [40], or predicting the risk of alcohol-related problems [45]. These behaviors, taken together with genetic evidence [42,43], suggest that sweet liking phenotype is an indicator of heritable dysfunction of the brain reward system [46,47]. Increased brain reward system activity is one proposed mechanism by which insufficient sleep can lead to excess energy intake [29]. Therefore, insufficient sleep may differentially increase brain reward function in sweet likers compared to other phenotypes. No study to date has investigated whether there is a relationship between insufficient sleep and sweet liking phenotype (SLP).

Due to reported differences in brain reward processing of sucrose compared to non-nutritive sweeteners (NNS) [48], sweetener type is an important factor to consider when examining the effect of sleep on hedonic response to sweet taste. While nutritive and NNS activate the same taste pathways in the brain, NNS have been shown to activate key reward centers (anterior insula, striatum and anterior cingulate) less than sucrose and fail to activate dopaminergic midbrain areas at all [48]. Given that increased brain reward sensitivity is a well-supported mechanism by which insufficient sleep is linked to increased energy intake, it stands to reason that insufficient sleep may differentially impact taste perception of nutritive and NNS sweeteners. Further, individual differences in hedonic response to sweet taste from NNS may not align with hedonic response to sucrose and, therefore, must also be considered separately. In addition, the effects of sweetener type on SLP have been explored almost exclusively by using nutritive sweeteners (e.g., [38,40,44,49]), so it is unclear if NNS will also show distinct liking phenotypes. To our knowledge, only one study has examined SLPs using an NNS and reported similar phenotypes in stevia [50]. Others have reported that roughly-equal sweet concentrations of NNS and sucrose are preferred similarly in healthy people [51], and that sweet tastes, whether from nutritive or NNS, stimulate higher order reward regions of the brain [52]. Therefore, it is likely, though unconfirmed, that sweet liking phenotypes extend to other sweeteners. 

The primary objective of the current study was to determine if sleep curtailment resulted in changes in preferred sweetener concentration and sweet taste intensity perception of sucrose and sucralose after sleep curtailment. A secondary objective was to determine if there is a relationship between SLP and insufficient sleep. Sucralose was selected as a representative NNS due to having a similar taste profile compared to sucrose and less off-flavor compared to other NNS [53,54]. Given the current psychophysical and behavioral evidence, it was hypothesized that insufficient sleep would result in an increase in sucrose preference and an increase in sucrose sweet liking at each sweetness level over a range of concentrations. While we expected similar findings for sucralose, we also hypothesized that the increase in liking after curtailment would be less pronounced in sucralose given the differences in brain response between the two sweeteners. Further, it was hypothesized that fundamental SLP classifications would exist for sucralose, and that sweet likers would be more susceptible to changes in sweet taste perception compared to other SLPs.

## 2. Materials and Methods 

The study protocol was approved by the Human Research Protection Program at Michigan State University (East Lansing, MI, USA). Written informed consent was obtained from all participants prior to testing.

### 2.1. Participants

Non-obese participants (BMI < 30.0 kg/m^2^) of any race or ethnicity between the ages of 18 and 45 with no diagnosed sleep conditions who normally slept 7–9 h per weeknight and had a regular weekday bedtime were eligible to participate in the study. Additionally, each participant was provided with a sample of the highest concentration of sucralose (0.094% weight/volume (% *w*/*v*)) to screen for bitterness sensitivity. While sucralose does not typically display high levels of bitterness [52], individuals who are highly sensitive to bitterness [40] may find it difficult to evaluate sucralose samples for sweetness. Participants who reported tasting any bitterness were not eligible for the study. Three people who were otherwise eligible were excluded for this reason. 

### 2.2. Study Timeline

Participants were required to attend an initial consent visit where the study administrator confirmed that each participant met the eligibility criteria for the study. After the consent visit, each participant visited the sensory laboratory for testing twice, once after a habitual night of sleep and once after a curtailed night of sleep, with at least 7 days between each visit. The second laboratory visit was required to take place on the same weekday and time (±30 min) as the first visit. Participants were randomly assigned to the sleep condition (habitual or curtailed) they would undergo first. Sleep time was centered to split the curtailment equally; that is, if the curtailment was 2 h, the participant was instructed to go to bed 1 h later and wake up 1 h earlier. To allow for community-dwelling conditions, participants were not instructed to follow any specific guidelines regarding the usage of time gained by curtailment. Centering the curtailment was designed to minimize circadian rhythm effects while still inducing sleepiness [55]. Curtailment was based on participants’ self-reported habitual bed and wake times. Partial sleep curtailment was selected because it represents a modest reduction in sleep that is more representative of community-dwelling conditions compared to total sleep deprivation [55]. Participants were tested as close to their wake-time as possible between 07:00 and 10:00 on any weekday (Monday–Friday) for sensory testing. These time slot options were selected to accommodate a range of possible habitual bed and wake times. 

### 2.3. Consent Visit

During the initial consent visit, eligible participants completed the Pittsburgh Sleep Quality Index (PSQI), Perceived Stress Scale (PSS), and the General Food Craving Questionnaire–Trait version (G-FCQ-T) and demographic questions. The PSQI [56], PSS [57], and G-FCQ-T [58] are validated questionnaires that were selected to assess subjective sleep, perceived stress, and general food craving traits, respectively. The PSQI measures subjective sleep quality and duration during the past month, and PSQI scores equal to five or greater indicate possible disordered sleep [56]. PSQI scores were measured to screen out participants with disrupted sleep in the past month who may not believe or be aware that they have disrupted sleep. The PSS measures perceptions of stress during the past month [57]. Chronic stress is associated with undesirable changes in sleep architecture [59]. PSS was measured to confirm that participants were not experiencing unusual chronic stress. The G-FCQ-T is a 21-item questionnaire which involves participants indicating the degree to which each item is generally true for them on a 6-point Likert scale ranging from 1 (never or not applicable) to 6 (always). These items are divided into subscales which measure nine dimensions of food cravings [60]. Food craving traits were measured as they may moderate reward sensitivity [61], and thus may aid in interpretation of findings. Height was measured using a stadiometer (HM200P, Charder, Taichung, Taiwan) and weight, body mass index (BMI), and percent body fat (%BF), were assessed using a bioelectrical impedance scale (TBF-400, Tanita, Arlington Heights, IL, USA). 

Participants were also trained to operate the Zmachine (General Sleep, Columbus, OH, USA) during the consent visit. The Zmachine records a single channel (A_1_–A_2_) of electroencephalography (EEG) and uses an automated scoring algorithm to differentiate between light sleep (LS), slow wave sleep (SWS), rapid eye movement (REM) sleep and waking states. When the performance of the Zmachine was compared to PSG, an overall kappa agreement of 0.72, indicating substantial agreement, was reported [62]. Participants were told to wear the Zmachine at least 30 min before the predetermined bedtime to ensure compliance with the assigned protocol. Finally, participants were instructed to not eat or drink anything other than water between their wake time and their scheduled sensory testing appointment. 

### 2.4. Laboratory Visits

The procedure for each of the two test visits was identical. Upon arriving at the lab after a night of sleep recording, the EEG data from the previous night’s sleep was immediately uploaded to the Zmachine data viewer, and the participant was asked to confirm that the data matched their own recollection of the previous night. If there was substantial data loss or evidence of machine malfunction (such as disagreement >30 min between reported machine and participant wake-time), the recording was reattempted after 7 days. Prior to beginning sensory testing, participants were asked to take a “Hydrogen Breath Test” by blowing into a metalized bag with a valve to ensure they had fasted. This procedure was a strategy used to encourage participants to adhere to the fasting instructions. The samples were not analyzed, and participants were told the true purpose of the “Hydrogen Breath Test” after completion of the study.

Prior to tasting any stimuli, participants self-administered a series of questionnaires including the Karolinska Sleepiness Scale (KSS), the Positive Affect-Negative Affect Schedule (PANAS), the General Food Craving Questionnaire-State version (G-FCQ-S), and a simple 100 mm visual analog scale (VAS) to measure hunger with “Extremely Hungry” (0) and “Extremely Full” (100) labels. The KSS [63], PANAS [64], and G-FCQ-S [58] are validated questionnaires used to measure sleepiness, affect, momentary food cravings, respectively. KSS is a 10-point category scale ranging from “Extremely alert” (1) to “Extremely sleepy, can’t keep awake” (10) [65]. The KSS was used to determine the effectiveness of the curtailment treatment. The PANAS was used to assess affect changes across the treatment conditions and is scored between 10 and 50 for both positive affect, which represents how much a person feels enthusiastic, active, and alert, and negative affect, which represents the extent to which a person feels anger, contempt, guilt, fear, and nervousness, separately (50 being more negative or more positive) [64]. Positive affect has been found to shift after a night of insufficient sleep [66] and, therefore, was measured to aid in the interpretation of findings. The G-FCQ-S contains 15 items which participants indicate on a 5 point Likert scale, ranging from “ Strongly disagree” (1) to “Strongly Agree” (5), the extent to which they agree with each item “right now, at this very moment”. The G-FCQ-S can be subdivided into 5 subscales which represent different dimensions of momentary food craving [60]. Craving states have been found to be associated with sleep duration [31] and, therefore, were measured to aid in the interpretation of findings. The VAS used to measure hunger has been shown to be a sensitive measure of hunger [67] and was used to assess whether fasting was effective in controlling for hunger.

To assess subjective sleep quality, participants answered four questions regarding their recollection of the previous night’s sleep. There are currently no validated questionnaires available for assessing previous night’s subjective sleep quality. Therefore, questions were developed to measure some dimensions of subjective sleep quality for the purpose of assessing whether the curtailment or Zmachine altered subjective sleep quality. The four questions were: “How much sleep did you obtain last night?” (1: Far less than I needed, 5: Far more than I needed), “How deeply did you sleep last night?” (1: Extremely shallow, 5: Extremely deep), “How would you rate the quality of your sleep last night?” (1: Poor, 5: Excellent), and “Compared to an average night of sleep, how comfortable were you when sleeping last night?” (1: Far less than an average night, 5: Far more than an average night). Additionally, a composite score of these questions was used to represent overall subjective sleep quality. 

### 2.5. Development of Iso-Sweet Stimuli

While several studies have developed iso-sweet stimuli between sucrose and non-nutritive sweeteners, none have extended into the concentration range needed to assess typical human sweetness preference with sucralose [68,69]. To compare hedonic response to sweetness across sweeteners, it was necessary to ensure that the concentrations of the two sweeteners were comparable. Thus, a preliminary study aimed at identifying iso-sweet concentrations of sucralose and sucrose was conducted per the methods of Reis, et al. [68]. Briefly, 100 participants assessed the relative sweetness of a range of concentrations of sucralose (0.005% *w*/*v*–0.16% *w*/*v*, *n* = 50) and sucrose (3% *w*/*v*–36% *w*/*v*, *n* = 50) using magnitude estimation with a fixed reference (12% sucrose). From these data, Steven’s power functions were produced and used to select concentrations of sucralose equivalent to the 3%, 6%, 12%, 18%, and 24% *w*/*v* sucrose. These concentrations were adapted from the Monell forced choice paired comparison protocol [70] used in the preference testing portion of the experiment (see below). Equivalent sucralose concentrations were found to be 0.004%, 0.011%, 0.032%, 0.06% and 0.094% *w*/*v*, respectively. 

### 2.6. Sensory Evaluation 

All sensory data was collected using RedJade Sensory Software (RedJade, Redwood Shores, CA, USA) at the Michigan State University sensory laboratory. All samples were served at room temperature in 10 mL quantities using 30 mL plastic soufflé cups. Participants wore nose-clips during all tastings. Additionally, participants were instructed to taste the whole sample and expectorate. The sensory evaluation consisted of two tasks; preference testing and liking evaluation. The two tasks were carried out first with sucrose solutions and then again with sucralose solutions of equal sweetness. This was done to reduce any possible effect of lingering sucralose aftertaste on sucrose taste perception [53]. 

### 2.7. Preference Testing

A modified version of the Monell forced choice paired comparison protocol [70] was used for preference testing. While the original Monell procedure used a wider range (3–36% *w*/*v*) of sucrose concentrations, at concentrations of sucralose equivalent to 36% *w*/*v* sucrose, the risk of bitter taste impairing sweetness evaluation increases [69]. However, in order to measure preference using a forced choice paired comparison, it is necessary to have at least five clearly distinguishable levels of sweetness while maintaining a mid-point that is close to the average sweetness liking seen in healthy populations [70]. If the range is too small, sweet likers could select the highest sweetness level every time, making it impossible to measure changes. Thus, the two highest concentrations from the Monell protocol, 24%, and 36% *w*/*v*, were reduced to 18% and 24% *w*/*v*. In a preliminary triangle test (*n* = 15), participants were able to discriminate 18% and 24% *w*/*v* sucrose (*p* < 0.05). The modification allowed for the avoidance of off tastes at high concentrations while maintaining the efficacy of the protocol. Aside from the modifications to the range of sweetness, the Monell protocol was followed. Participants were given two concentrations of suprathreshold sweetener and asked to point to the solution which they liked more. Participants rinsed with purified water between tasting each solution in the pair and between each set of pairs. Based on their selection, a second pair containing the concentration they previously selected and an adjacent concentration were presented until they selected the same solution twice in a row. The protocol was repeated twice, first with the lower concentration presented first and second with the higher concentration presented first. The geometric mean of the % *w*/*v* preferred sweetener concentration is reported as the “preferred sweetener concentration”.

### 2.8. Evaluation of Sweetness Liking

Sweetness liking was assessed by presenting a range of different concentrations of sweetener solutions identified with three-digit blinding codes in random order. Due to interest in changes in liking slope and SLP, eight increasing concentrations were used to ensure patterns of liking would be unambiguous. The sweetener concentrations included 3%, 6%, 9%, 12%, 15%, 18%, 21% and 24% *w*/*v* sucrose and 0.004%, 0.011%, 0.020%, 0.032%, 0.045%, 0.060%, 0.075%, 0.094% *w*/*v* sucralose. Participants were asked to rate their liking of each solution on a 15 cm VAS scale with anchors at 0 (dislike extremely), 7.5 (neutral) and 15 (like extremely). Additionally, participants were asked to rate how intensely they perceived the sweetness to be on a 15 cm VAS scale with anchors at 0 (not at all intense) and 15 (extremely intense). Following the tasting of a solution, there was a 45 s forced wait period in which the participant was required to rinse three times with purified water. 

### 2.9. Statistical Analysis 

Data analysis was completed using SAS version 9.4 (SAS Institute, Cary, NC., USA). Findings were considered statistically significant if *p* < 0.05 in all analyses, and data are presented as the mean ± standard deviation unless otherwise stated. Liking scores were plotted against sweetener concentration, and the best fit linear function was calculated in Excel (Microsoft, Redmond, WA, USA) and used to determine the “Liking Slope” variable used throughout the study. 

A mixed model was used to compare the main effects of sleep curtailment and the interaction effects between SLP (*n* = 2, sweet likers and non-likers, see “*Sweet Liking Phenotypes*” section below), sweetener type (*n* = 2, sucrose and sucralose) and sleep curtailment (*n* = 2, habitual and curtailed sleep) on preferred sweetener concentration and sweet liking slope. Participant and interactions between participant and the main effects were included as random factors. Sequence (curtailed or habitual night first) and period (first or second visit) were initially included to determine whether there were significant carry-over effects. No significant sequence or period effects were observed for preferred sweetener concentration (sequence: *p* = 0.44, period, *p* = 0.84) or liking slope (sequence: *p* = 0.25, period, *p* = 0.20) and therefore were not used in any further analysis. Tukey’s correction was used for multiple mean comparisons in all cases. Paired data collected from participants after a habitual or curtailed night’s sleep, such as PANAS scores or hunger rating, were analyzed using paired t-tests and corrected for multiple comparisons using false discovery rate (FDR) with a threshold of *q* = 0.05, which has been used previously to reduce the risk of type-1 error in psychophysical studies [23,71]. To provide additional evidence regarding comparisons between the sweeteners, associations between the preferred sweetener concentration of the two sweeteners were assessed using Pearson correlations. Pearson correlations were also used to assess the relationship between participant baseline measure of PSQI, G-FCQ-T, PSS and preferred sweetener concentration (see participants below). Hierarchical cluster analysis (HCA), an objective strategy for determining SLPs that is recommended as the standard for sweet liking classification [37], was conducted in XLstat (Addinsoft, Paris, France) using the eight liking scores across the range of concentrations of each sweetener in order to classify participants into SLPs [36]. In order to compare sucrose and sucralose preference, sucralose preference (% *w*/*v*) was converted to sucrose preference equivalents using the power functions discussed above to produce a single dependent variable. 

## 3. Results

### 3.1. Participants

Participant demographics are reported in Table 1. Forty participants with BMI measurements of <30.0 kg/m^2^ (without obesity) completed the study. Participants were majority white (*n* = 26) and female (*n* = 27). All participants had a PSQI score ≤5. ANOVA was used to assess interactions between sleep treatment and sex. Sex did not show a significant main effect and there was no significant interaction between sex and sleep treatment for any sensory measure (*p* > 0.05). Data for both sexes were therefore pooled. Anthropometric measurements as well as PSQI, G-FCQ-T and PSS scores were not correlated with preferred sucrose or sucralose concentration and therefore were not utilized in further analysis (*p* > 0.05).

### 3.2. Summary of Curtailment

Sleep curtailment resulted in expected changes in sleep architecture, sleepiness, and subjective evaluation of the previous night’s sleep. A 35.3% reduction in TIB resulted in reductions in TST, LS, REM and SWS duration (*p* ≤ 0.001 for all) (Table 2). These changes in sleep architecture and duration resulted in an increase in sleepiness, as evidenced by the increase in KSS score (*p* < 0.001). Participants rated the previous night’s sleep as less than needed after curtailment but did not perceive the “deepness”, “quality” or “comfort” to be significantly different than the habitual night. Sleep quality was rated slightly above “about average” on both the habitual and curtailed nights. 

### 3.3. Summary of Affect, Cravings, and Hunger

Curtailment did not result in changes in hunger, food cravings, or negative affect (Table 3). Curtailment resulted in a decrease in positive affect.

### 3.4. Sweet Liking Phenotypes

**Figure 1:**
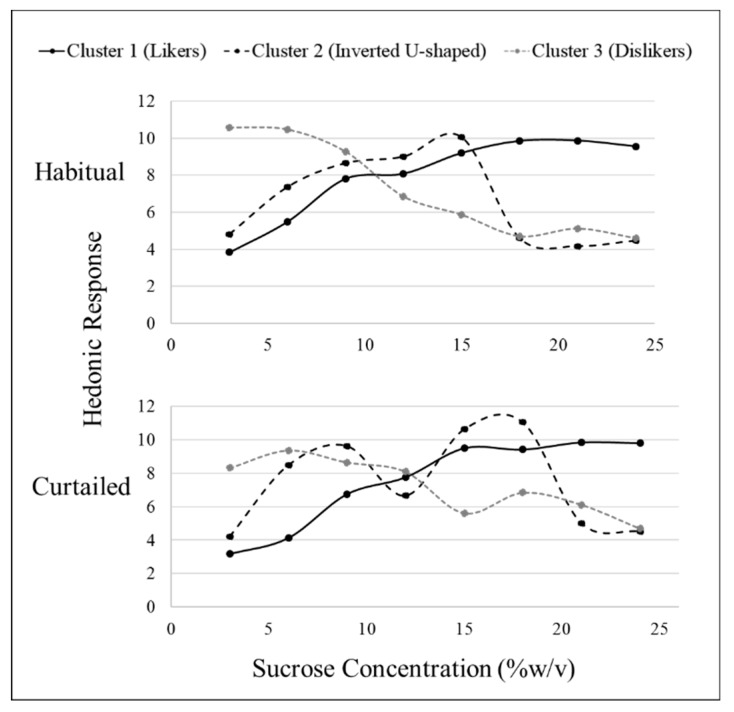
Hierarchical cluster analysis identified three sucrose sweet liking phenotypes based on sweet liking response using a 15 cm visual analog scale; however, patterns differed between habitual and curtailed nights. After the habitual night, cluster 1 (*n* = 25), cluster 2 (*n* = 6), and cluster 3 (*n* = 9) demonstrated the fundamental phenotypes of sweet liking. After the curtailed night, cluster 1 (*n* = 28) and cluster 3 (*n* = 8) retained the familiar fundamental patterns of liking; whereas, cluster 2 (*n* = 4) had a distorted pattern.

**Figure 2:**
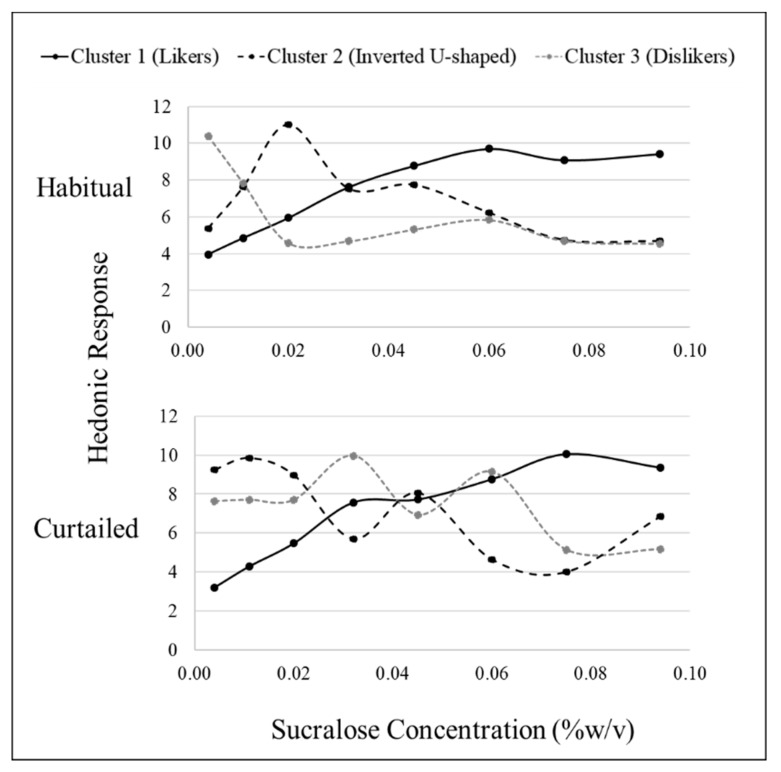
Hierarchical cluster analysis identified three sucralose sweet liking phenotypes based on sweet liking response using a 15 cm visual analog scale; however, patterns differed between habitual and curtailed nights. After the habitual night, cluster 1 (*n* = 24), cluster 2 (*n* = 10), and cluster 3 (*n* = 6) demonstrated the fundamental phenotypes of sweet liking. After the curtailed night, cluster 1 (*n* = 29) retained the familiar fundamental pattern of liking; whereas, cluster 2 (*n* = 3) and cluster 3 (*n* = 8) had distorted patterns.

Hierarchical cluster analysis revealed three fundamental clusters of SLPs for both sucrose and sucralose after habitual sleep (Table 4). After a habitual night’s sleep, each cluster presented a distinct pattern of liking (Figure 1 and Figure 2). Members of cluster 1, the largest cluster, increasingly liked the stimuli (“likers”) until leveling off at approximately 18% *w*/*v* sucrose or 0.06% *w*/*v* sucralose. Cluster 2 members displayed an inverted U-shape of liking ratings with maximum liking occurring at approximately 15% *w*/*v* for sucrose and 0.02% *w*/*v* for sucralose (“inverted U-shaped”). Members of cluster 3 rated increasing concentrations as decreasingly liked (“dislikers”) until leveling off at approximately 18% *w*/*v* sucrose or 0.06% *w*/*v* sucralose. Due to the small sample size, clusters 2 and 3 were combined for use within the “SLP” two level factor (sweet likers and sweet non-likers) in mixed models analysis. 

After a curtailed night of sleep, the fundamental phenotypes observed after a habitual night of sleep became less distinct. For sucrose, cluster 1 still showed an increase in liking until leveling off at the 18% *w*/*v* concentration and cluster 3 still showed a decrease in liking as concentration decreased. Cluster 2 no longer displayed a clear, fundamental pattern of response (Figure 1). For sucralose, cluster 1 still showed an increase in liking until leveling off at the 0.060% *w*/*v*. Clusters 2 and 3 lost the fundamental SLPs with patterns becoming distorted after sleep curtailment. The formerly inverted U-shaped pattern displayed in cluster 2 showed a bimodal pattern with vertices above and below the midpoint, and cluster 3, formerly displaying a disliking pattern, displayed a bimodal pattern in the opposite direction (Figure 2). After a habitual night’s sleep, 75% of participants had matching (i.e., in the same cluster) sucrose and sucralose liking phenotypes. After a curtailed night of sleep, 83% of participants had matching sucrose and sucralose liking phenotypes. The distribution of participants among the clusters, or how many participants were placed into each cluster, was not significantly different between the sweeteners, nor was member distribution between the clusters significantly modified after a curtailed night of sleep (Kolmogorov–Smirnov, *p* > 0.05).

### 3.5. Sweet Preference

**Figure 3:**
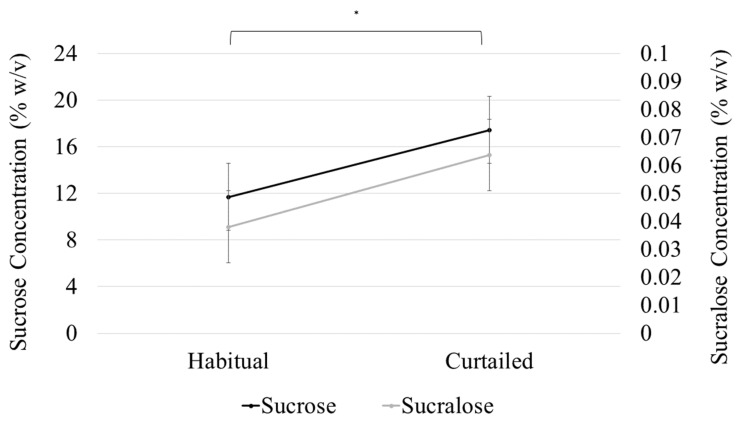
Sucrose and sucralose preferred concentration increased significantly (* *p* < 0.001) after sleep curtailment. Points represent preferred concentration and error bars represent standard error of the mean.

A model with sleep condition, sweetener type, SLP, and all interactions up to the tertiary level was used to analyze preferred sweetener concentration. The effect of sleep on preferred sweetener level was not related to SLP or sweetener types, as the interaction terms between sleep condition and both sweetener type (F(1,38) = 0.24, *p* = 0.62) and SLP (F(1,38) = 2.0, *p* = 0.164) were not significant. The interaction between SLP and sweetener type was not significant (F(1,38) = 0.02, *p* = 0.898), signifying that the difference in preferred sweetener concentration between the SLPs was not specific to either sweetener. The main effect of sleep condition on preferred sweetener concentration (F(1,38) = 130.8, *p* < 0.001) was significant, indicating a difference in preferred sweetener concentration after sleep curtailment (sucrose (M (difference) = 5.4% *w*/*v*, SD = 6.5); sucralose (M (difference) = 5.7% *w*/*v* sucrose equivalencies, SD = 6.7) (Figure 3). Preferred sweetener concentration was not different between the sweeteners, regardless of SLP or sleep condition; that is, preferred sucralose concentration (as sucrose equivalents) was not significantly different from preferred sucrose concentration after both habitual (12.7 sucrose % *w*/*v* vs. 11.7% *w*/*v* sucrose equivalents for sucralose) and curtailed sleep (18.1 Sucrose % *w*/*v* vs. 17.4% *w*/*v* sucrose equivalents for sucralose (sweetener main effect; (F(1,38) = 3.1, *p* = 0.086)). Sucrose and sucralose preferred concentrations were strongly and positively correlated (r = 0.8356, *p* < 0.001). The main effect of SLP on preferred sweetener concentration was significant (F(1,38) = 37.62, *p* < 0.001), indicating that preferred sweetener concentration differed between sweet likers and sweet non-likers (Table 5).

### 3.6. Sweet Intensity 

**Figure 4:**
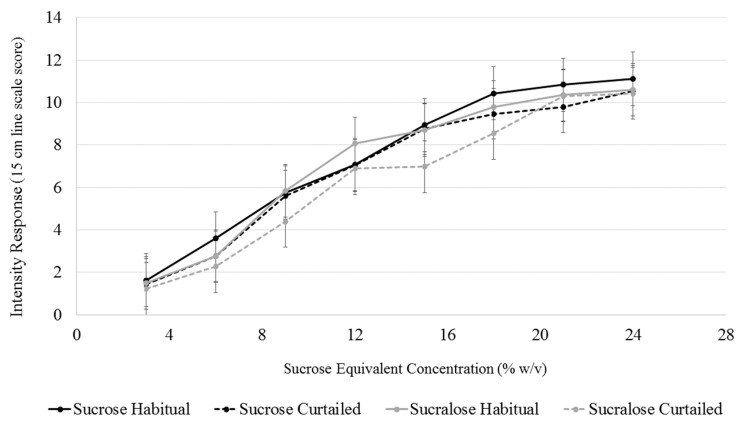
Comparison of intensity perception of sucrose and sucralose (in sucrose equivalents) after a habitual and curtailed night of sleep. No significant differences between the sweeteners after either sleep condition (*p* > 0.05). Error bars represent standard error of the mean.

A model with sleep condition, sweetener type, sweetener concentration, SLP, and all interactions up to the tertiary level was used to analyze explicit sweet intensity. Changes in sweet intensity perception at each concentration level for both sweeteners after sleep curtailment were assessed using the interaction terms between sleep condition, sweetener, and concentration level. The interaction term between sleep condition and concentration level was not significant (F(7,1245) = 0.81, *p* = 0.818), indicating that sleep curtailment did not alter intensity perception at any sweetener concentration. Further, the interaction term between sweetener concentration and sweetener type was not significant (F(7,1245) = 0.74, *p* = 0.640), signifying that the intensity of two sweeteners were not different at any concentration level (Figure 4). 

### 3.7. Sweet Liking 

A model with sleep condition, sweetener type, sweetener concentration, SLP, and all interactions up to the tertiary level was used to analyze sweet liking responses. Some comparisons between sweetness levels were significant after sleep curtailment, as the interaction term between sleep condition and sweetener concentration was significant for sweet liking (F(1,1245) = 2.1, *p* = 0.046). However, comparisons between like concentrations (for example, the comparisons between 3% *w/v* liking after a habitual and curtailed sleep) were not found to be significant during post-hoc testing (*p* > 0.05). Further, neither the sleep condition and sweetener type interaction (F(1,1217) = 0.17, *p* = 0.677), nor the tertiary sleep condition, sweetener type and sweetener concentration interaction (F(7,1245) = 0.59, *p* = 0.762) were significant, signifying no difference in liking after sleep curtailment between the two sweeteners after sleep curtailment. 

**Figure 5:**
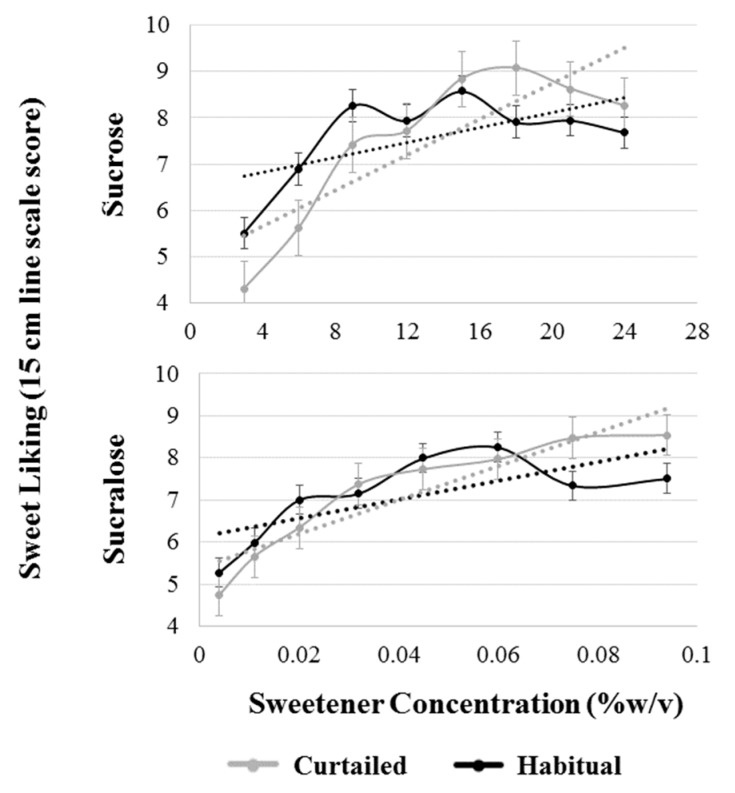
Comparison of patterns of sweet liking of sucrose and sucralose after a habitual and curtailed night of sleep for all participants. Black dotted lines represent the best fit linear slope for the pattern of liking after the habitual night; gray dotted lines represent the best fit linear slope for the pattern of liking after the curtailed night. The habitual liking slope and curtailed liking slope are significantly different for sucrose (*p* = 0.001), but not sucralose (*p* = 0.129). Error bars represent standard error of the mean.

A model with sleep condition, sweetener type, SLP, and all interactions up to the tertiary level was used to analyze sweet liking slope. The interaction term between sleep condition and sweetener type was significant (F(1,38) = 4.97, *p* = 0.032), indicating a differential effect of sleep curtailment on the two sweeteners. Post-hoc testing revealed a significant increase in the steepness of sucrose liking slope (*p* = 0.001) but not sucralose liking (*p* = 0.129) (Figure 5). Sucrose liking slope shifted from 0.08 to 0.19 increase in hedonic response per 1% *w/v* increase in sucrose concentration after sleep curtailment; whereas, sucralose slope moved from 0.11 to 0.18 sucrose equivalent rate of change. 

## 4. Discussion

The primary objective of this study was to characterize the impact of modest sleep curtailment on chemosensory function and hedonic perception of sweetness from sucrose and sucralose. It was hypothesized that a 33% reduction in sleep duration would result in a shift toward increased liking and preference for sweetness from sucrose and, to a lesser degree, from sucralose. Sleep curtailment resulted in the hypothesized increase in preferred sweetener concentration in both sucrose and sucralose. However, sleep curtailment did not result in a clear shift towards increased liking of all levels of sweetness. Rather, a complex series of changes in hedonic perception of sweetness that resulted in a steeper pattern of liking as sweetness increased in sucrose, and no significant difference, but a similar pattern, in sucralose liking was observed. The change in slope steepness occurs in such a way that individual levels of sweetness, when compared directly, are not significantly different from one another. This may explain why others who have previously examined the effect of sleep curtailment concluded that sleep does not influence hedonic perception of sweetness [71], as patterns of liking were not analyzed. Further, it was hypothesized that changes in liking would occur independently of changes in taste intensity perception. In agreement with our hypothesis, no changes in sucrose or sucralose intensity perception were observed after sleep curtailment. Finally, it was hypothesized that fundamental SLPs would exist for sucralose. Participants were grouped by SLPs using HCA. Sucrose phenotypes were similar to sucralose phenotypes after a habitual night of sleep, with 75% of participants belonging to the same SLP for both sucrose and sucralose after the habitual night, and commonly reported (so-called “fundamental”) phenotypes were present. 

To our knowledge, this is the second attempt to classify sweet liking patterns using a non-nutritive sweetener [50], and the first using sucralose. Whether a participant was classified as a liker (cluster 1) or a non-liker (clusters 2 and 3) was predictive of preferred sweetener concentration for both sweeteners. While almost all of the work exploring sweet liking phenotypes has been done using sucrose, that these phenotypes are also present when sucralose is used as the stimulus and when stevia is used [50] suggests that these classifications extend to other sweeteners. While there appear to be some cases of individual variability, where a sucrose disliker was not a sucralose disliker, in general, the phenotypes were relatively stable across sweeteners.

While it was hypothesized that there would be a shift in liking so that all levels of sweetness would show an increased hedonic response after a night of sleep curtailment, the findings suggest a more complex relationship where the pattern of liking was altered so that the slope of the best-fit linear function of the hedonic response–concentration plot became significantly steeper after sleep curtailment. The change in pattern suggests a shift in hedonic responses so that higher concentrations of sweetness are more liked and lower concentrations are less liked after sleep curtailment, which, taken together with reported changes in desire for sweet and high-carbohydrate foods [13,17], could contribute to the association between insufficient sleep and excess energy intake. The notion that higher concentrations of sweetness are more liked after sleep curtailment is further supported by the significant increase in preferred sweetener concentration for both sweeteners. The increase in steepness of the slope may also be driven partially by a decrease in liking of lower concentrations of sucrose. The significant shift in slope of the liking function suggests that low concentrations are generally less liked after a curtailed night of sleep. Given that sleep deprivation has been associated with increased neural and behavioral reactivity to both negative and positive experiences [26], it is possible that an increase in liking of highly sweet solutions and a decrease in liking of less sweet solutions occurs simultaneously. 

The two sweeteners were not perceived as differently intense or pleasurable. Under normal conditions, adults have been shown to prefer approximately equally sweet concentrations of sucrose and sucralose [51], which is in agreement with the data presented in the current study. Importantly, average sweetness intensity for both sweeteners was not significantly different from one another at any of the sweetness levels, indicating that, at each level of sweetness, the two sweeteners were approximately iso-sweet, as designed. Despite previous research suggesting that sucralose and sucrose may differentially stimulate reward processing centers in the brain [48], participants in the current study preferred equivalent concentrations of sweetness between the two sweeteners (as measured by sucrose equivalency). However, the change in participants’ sucralose liking over the range of concentrations after curtailment, while similar in shape to sucrose, was not significant. While not statistically significant, the similarities in the shapes of the two curves after curtailment suggest that a similar modification of patterns of sweet liking may be occurring, albeit to a lesser degree, as hypothesized. Other than the magnitude of the change in slope, sleep curtailment generally did not appear to differentially impact sweet taste perception of the two sweeteners. However, we cannot conclude that the two sweeteners were equally affected by sleep curtailment due to the lack of statistically significant change in the slope of sucralose liking. 

A preferential increase in sucrose liking after sleep curtailment compared to sucralose could have important dietary implications. Sucrose preference might be increased by insufficient sleep due to alteration in dopaminergic midbrain function; whereas, preference for an NNS, such as sucralose, may be less affected due to sucralose’s lack of midbrain interaction [48]. If this is the case, sleep curtailment could increase the palatability of sucrose while leaving sucralose palatability unchanged. However, the increase in palatability of high concentrations of sucrose may lead to excess energy intake, suggesting that sucralose might be a better sweetener option for habitually short sleepers. Alternatively, sucralose may be relatively sub-optimal at satisfying sweet cravings compared to sucrose in individuals who had an insufficient previous night’s sleep, driving increased consumption. Therefore, more work is needed to assess differences in hedonic response between nutritive and NNS after sleep curtailment and how these changes influence dietary intake, if at all.

While it was hypothesized that sweet likers might be more susceptible to changes in sweet liking after sleep curtailment, the data did not support this hypothesis. Both sweet likers and non-likers showed an increase in preferred sweetener concentration for both sweeteners after sleep curtailment. However, it should be noted that the absolute increase in preferred sucrose concentration is similar for all of the clusters; therefore, having a low habitual sweetness preference may still be protective against the effects of sleep curtailment when considering how these changes may manifest to alter food choice. For example, a sweet liker, who, after sleep curtailment, prefers sucrose concentrations as large as 17% *w*/*v*, may be at higher risk for selecting a high calorie sweetened foods compared to a non-liker who still only prefers between 10 and 12% *w*/*v* sweetener concentration after sleep curtailment. However, sweet taste perception is not always predictive of dietary intake [72], and therefore, the effects of these perceptual changes on food choice cannot yet be determined until more is understood about how momentary taste preferences inform eating behaviors.

Whether the present results pertain to individuals who are chronically sleep curtailed is uncertain. The current study used an acute sleep curtailment protocol and recruited participants who reported that they typically met suggested sleep duration guidelines. There are no studies that have examined relationships between SLP and habitual sleep habits. There is evidence that neurobehavioral effects of sleep curtailment are cumulative [73], and therefore, it is possible that chronic sleep curtailment is related to preference for higher sweetness or expressed SLP. However, individuals who experience chronically curtailed sleep (habitual short sleepers) are likely distinct from those who generally meet sleep recommendations and are then acutely sleep curtailed as in the current study. While previous research reports an inverse linear relationship between previous night sleep duration and next day preferred sucrose concentration [22,23], one study reported that the preferred sweetness concentration of habitual short (sleeping 7 or fewer h/night) and habitual long sleepers (sleeping >7 h/night) responded differently after a night of sleep curtailment [32]. Preferred sweetness concentration increased among habitual long sleepers after sleep curtailment but not for habitual short sleepers. SLP was not measured, and preferred sweetness concentration between the two groups was not directly compared. Whether SLP is distorted after a night of curtailment in habitual short sleepers or whether SLP classification changes after a night of longer sleep are questions worthy of further study. 

Sleep curtailment resulted in a significant decrease in positive affect and no change in negative affect. Positive affect can be defined as a state of pleasurable engagement with the environment that elicits feelings, such as happiness or joy [74]. Negative affect can be defined as a state of unpleasant engagement with the environment that elicits feelings, such as anxiety or anger [75]. Positive and negative affect are thought to be statistically independent [76]. In agreement with our findings, previous literature has reported a decrease in positive affect without changes in negative affect after a night of sleep curtailment [77,78]. It is important to note that changes in negative affect are have been reported when participants were totally sleep deprived, but not after partial sleep curtailment, [79] and that the modest curtailment used in the current study may not have been large enough to elicit changes in negative affect. The difference in positive affect between the sleep conditions may play a role in the differences in hedonic response to the sweet stimuli. One study reported that positive affect was associated with increased acceptance of generally less preferable flavors, suggesting that less-preferable stimuli become more acceptable when in a state of high positive affect [80]. Higher positive affect after a habitual night of sleep may partially explain the shift in the sweet liking slope, as sweet likers with higher positive affect may rate less preferable low concentrations more favorably. However, our participants were clearly not in a “state of high positive affect”, given the mean (23.8) is lower than normative momentary positive affect measured using the PANAS (29.7) [64]. It is not clear if increased liking of less preferable flavors is linearly associated with positive affect or if increased liking only occurs after a threshold of positive affect is reached. 

### Strengths and Limitations 

The strengths of this study include the randomized crossover design with testing sessions held one week apart on the same day within 30 min of the previous session under fasted conditions. Another strength is the use of the Zmachine EEG to collect objective at-home sleep data from participants. The Zmachine allowed for the confirmation of adherence to the prescribed sleep curtailment. Limitations of this study include possible fatigue effects from the large sample tasting load per lab visit. To minimize this, breaks between trials were instituted. Further, the range of sweetness levels used may not have been large enough to fully capture changes in preferred sweetener concentration after sleep curtailment, as evidenced by participants who selected the highest level of sweetness after a habitual (sucrose *n* = 4, sucralose *n* = 4) or curtailed night of sleep (sucrose: *n* = 11, sucralose *n* = 12) in at least one of the two trials during sweet preference testing. Participants in this study were non-obese per BMI, although %BF was slightly elevated in some individuals; thus, generalizing the findings to individuals with elevated adiposity should be avoided until further testing can be performed.Several limitations regarding the sleep curtailment strategy used are also present. While participants reported healthy subjective sleep (PSQI <5) prior to taking part in the study, sleep duration was not measured for the days prior to the recording night, and, therefore, we cannot rule out the possible effect of cumulative nights of insufficient sleep [81]. While no order or sequence effects were observed, participants were not habituated to the Zmachine prior to use, and therefore, first-night effects caused by experiencing the Zmachine for the first time may have occurred. Unpublished data from our lab revealed no differences in objective sleep measures between the first and second nights of Zmachine use in a population of 41 females who claimed to habitually meet sleep recommendations, so this appears to be of limited concern but is still worth noting. Despite curtailment occurring around the habitual sleep midpoint, depending on how participants chose to use the additional time gained through sleep curtailment, circadian rhythm disruption could still have occurred. Finally, sweet taste alone was measured using prototypical tastants in water, and therefore, it is unclear how these sleep curtailment-induced changes manifest, if at all, when complex foods with multiple sensory attributes are consumed.

## 5. Conclusions

Healthy participants who were not obese had increased preference for sweetness and fundamental SLPs were distorted after a night of modestly curtailed sleep. These findings suggest that increased energy intake related to insufficient sleep may be moderated by altered hedonic and chemosensory perception. While the shift in the slope of the liking of sucralose was similar in appearance to sucrose, there was, statistically, no change in sucralose liking slope, which could be related to differential brain processing of the two sweeteners after sleep curtailment. Finally, significant changes in sweet taste perception after modest sleep curtailment suggest that it may be necessary to control for sleep in food sensory studies. However, future work is needed to determine whether perception of more complex food stimuli is altered after a curtailed night of sleep. Finally, future studies should aim to determine temporal factors involved in the observed relationship, such as the longevity of the effects and the effect of chronic sleep curtailment.

## Figures and Tables

**Table 1:** Anthropometric and demographic summary.

Sex	N	%
Male	13	32%
Female	27	67%
Race	n	%
White	26	65%
Asian	12	30%
Other/More than 1	2	5%
Anthropometrics	Mean ± SD	Range
Body mass index (kg/m^2^)	22.9 ± 3.0	18.5–29.7
Body fat (%)	22.3 ± 7.9	9.9–35.5
Age (y)	23.8 ± 4.6	18–37
Traits/Habits (score)	Mean ± SD	Range
General food craving questionnaire-trait version	51.3 ± 17.2	22–89
Perceived stress scale	11.3 ± 4.4	3–21
Pittsburgh Sleep Quality Index	3.3 ± 1.4	0–5

**Table 2:** Summary of objective and subjective sleep measures.

		Habitual	Curtailed	% Reduction	*p*-Value	*q*-Value
Objective Sleep Measures (h)	Time in bed	8.2 ± 0.7	5.3 ± 0.7	35.30%	<0.001	<0.001
	Total sleep time	7.0 ± 0.8	4.5 ± 0.8	36.00%	<0.001	<0.001
	Light sleep	3.6 ± 0.7	2.0 ± 0.6	44.20%	<0.001	<0.001
	REM sleep	1.9 ± 0.5	1.1 ± 0.3	40.40%	<0.001	<0.001
	Slow wave sleep	1.6 ± 0.3	1.3 ± 0.4	16.70%	<0.001	<0.001
Sleepiness (10 pt)	Karolinska Sleepiness scale	3.9 ± 1.6	5.5 ± 1.8		<0.001	<0.001
Subjective Previous Night’s Sleep Measures (5pt)	Subjective Sleep Composite	12.8 ± 2.1	10.9 ± 2.6		<0.001	<0.001
	How much sleep did you obtain last night?	2.9 ± 0.6	1.5 ± 0.6		<0.001	<0.001
	How deeply did you sleep?	3.7 ± 0.9	2.6 ± 0.9		0.491	0.534
	How would you rate the quality of your sleep	3.4 ± 0.7	3.1 ± 1.3		0.209	0.256
	Compared to an average night, how comfortable were you when sleeping last night?	2.8 ± 0.6	2.7 ± 0.7		0.711	0.711

All objective sleep measures were significantly reduced after sleep curtailment. Karolinska Sleepiness Scale (KSS) (for which greater scores indicate decreased alertness) was significantly higher and composite subjective previous night’s sleep score was significantly lower after sleep curtailment, indicating that the curtailed night of sleep was perceived by participants to be of shorter length compared to a habitual night, resulting in decreased alertness the following morning. FDR correction did not change the significance of any comparisons.

**Table 3:** Summary of state-dependent measures.

Measure	Factor	Habitual	Curtailed	*p*-Value	*q*-Value
Hunger	Hunger (100 mm VAS)	66.0 ± 15.6	69.6 ± 15.1	0.193	0.248
G-FCQ-S (0–15 per factor)	Total	42.9 ± 10.8	46.3 ± 10.8	0.071	0.159
	F1-Desire to Eat	8.3 ± 3.0	9.0 ± 3.1	0.189	0.248
	F2-Anticipation to positive reinforcement	8.8 ± 3.2	10.0 ± 3.1	0.022	0.099 ^a^
	F3-Anticipation to negative reinforcement	9.8 ± 2.9	10.5 ± 2.3	0.104	0.188
	F4-Obsessive preoccupation	6.4 ± 2.2	6.8 ± 2.5	0.347	0.390
	F5-Craving as a physiological state	9.7 ± 2.5	10.0 ± 2.9	0.534	0.534
PANAS	Positive Affect	23.8 ± 8.7	20.5 ± 7.1	0.005	0.040
	Negative Affect	13.8 ± 5.3	15.0 ± 6.0	0.050	0.150

Positive affect was significantly decreased after sleep curtailment; whereas, hunger, food craving, and negative affect were not. Larger numbers indicate a greater response. For example, positive affect is higher (23.8) after a habitual night compared to a curtailed night (20.5). ^a^ FDR correction resulted in the comparison between F2 of the G-FCQ-S before and after sleep curtailment no longer showing significance. Abbreviations: VAS: Visual Analog Scale, G-FCQ-S: General Food Craving Questionnaire State Version, PANAS: Positive Affect Negative Affect Schedule, F1-5: General Food Craving Questionnaire State Version Factors 1–5.

**Table 4:** Distribution of members between sweet liking phenotypes.

Sweetener	Sleep Status	Sweet Likers (*n*)	Sweet Non-likers (*n*)	
			Inverted U-Shape	Sweet Dislikers
Sucrose	Habitual	25	6	9
	Curtailed	28	4	8
Sucralose	Habitual	24	10	6
	Curtailed	29	3	8

Sweet liking phenotype cluster membership distribution did not differ between the sweeteners.

**Table 5:** Comparison of preferred sweetener concentration after a habitual and curtailed night of sleep for each sweet liking phenotype (determined after a habitual night of sleep).

Habitual Cluster		Preferred Concentration (% *w*/*v*)			
		Sucrose		Sucralose	
		Habitual	Curtailed	Habitual	Curtailed
“Sweet Likers”	1 (Likers)	14.9 ± 4.4 *	17.5 ± 4.4 *	0.05 ± 0.02 *	0.08 ± 0.02 *
“Sweet Non-Likers”	2 (Inverted U-shape)	8.4 ± 5.3	12.4 ± 3.8	0.03 ± 0.03	0.04 ± 0.02
	3 (Dislikers)	5.1 ± 2.8	10.0 ± 1.9	0.01 ± 0.01	0.03 ± 0.02
	Non-likers Total	6.8 ± 4.1	11.4 ± 2.9	0.02 ± 0.02	0.03 ± 0.02

The main effect of sweet liking phenotype (SLP) for sweet preference was significant, indicating that sweet likers had a significantly higher preferred concentration for both sweeteners regardless of sleep status (* *p* < 0.001) compared to sweet non-likers. The *SLP by sweetener type* interaction for preferred sweetener concentration was not significant, signifying that preferred sweetness concentration did not differ by sweetener type. Sweet taste preference was not differentially effected by sleep curtailment, as the *SLP by sleep condition* interaction was not significant.
